# Dietary BMAA Exposure in an Amyotrophic Lateral Sclerosis Cluster from Southern France

**DOI:** 10.1371/journal.pone.0083406

**Published:** 2013-12-13

**Authors:** Estelle Masseret, Sandra Banack, Farid Boumédiène, Eric Abadie, Luc Brient, Fabrice Pernet, Raoul Juntas-Morales, Nicolas Pageot, James Metcalf, Paul Cox, William Camu, the French Network on ALS Clusters Detection and Investigation

**Affiliations:** 1 Ecologie des Systèmes Marins Côtiers, Unité Mixte de Recherche 5119, Institut de Recherche pour le Développement, Ifremer, Université Montpellier 2, Montpellier, France; 2 Institute for Ethnomedicine, Jackson, Wyoming, United States of America; 3 Net, Unité Mixte de Recherche 1094, Université de Limoges, Limoges, France; 4 Laboratoire Environnement Ressources/Languedoc Roussillon, Ifremer, Sète, France; 5 Unité Mixte de Recherche 6553, Eco-bio, Université de Rennes I, Rennes, France; 6 Ifremer-Laboratoire Environnement Ressource Languedoc-Roussillon, Sète, France; 7 Amyotrophic Lateral Sclerosis Center, Centre Hospitalier Universitaire Gui de Chauliac and Institut National de la Santé et de la Recherche Médicale U1051, Montpellier University 1, Montpellier, France; Macquarie University, Australia

## Abstract

**Background:**

Dietary exposure to the cyanotoxin BMAA is suspected to be the cause of amyotrophic lateral sclerosis in the Western Pacific Islands. In Europe and North America, this toxin has been identified in the marine environment of amyotrophic lateral sclerosis clusters but, to date, only few dietary exposures have been described.

**Objectives:**

We aimed at identifying cluster(s) of amyotrophic lateral sclerosis in the Hérault district, a coastal district from Southern France, and to search, in the identified area(s), for the existence of a potential dietary source of BMAA.

**Methods:**

A spatio-temporal cluster analysis was performed in the district, considering all incident amyotrophic lateral sclerosis cases identified from 1994 to 2009 by our expert center. We investigated the cluster area with serial collections of oysters and mussels that were subsequently analyzed blind for BMAA concentrations.

**Results:**

We found one significant amyotrophic lateral sclerosis cluster (p = 0.0024), surrounding the Thau lagoon, the most important area of shellfish production and consumption along the French Mediterranean coast. BMAA was identified in mussels (1.8 µg/g to 6.0 µg/g) and oysters (0.6 µg/g to 1.6 µg/g). The highest concentrations of BMAA were measured during summer when the highest picocyanobacteria abundances were recorded.

**Conclusions:**

While it is not possible to ascertain a direct link between shellfish consumption and the existence of this ALS cluster, these results add new data to the potential association of BMAA with sporadic amyotrophic lateral sclerosis, one of the most severe neurodegenerative disorder.

## Introduction

Amyotrophic lateral sclerosis (ALS) is one of the most severe neurological disorders with a median time of 36 months between onset and death [1]. The disease is characterized by progressive degeneration of both upper and lower motor neurons, but its origin, except for a subset of hereditary cases (4 to 8 % of total ALS cases), remains largely unknown. In the last 30 years, several environmental studies have pointed to the potential role of dietary exposure to β-methyl amino-L-alanine (BMAA) as a possible risk factor for ALS [2-6]. In the Middle Eastern deserts and in the Pacific island of Guam, BMAA exposure is associated with an elevated prevalence of sporadic ALS [7,8]. In Guam, it has been suggested that eating flying foxes as a delicacy along with feral animals and food products made of the starchy interior of the cycad seed all contribute to the BMAA found within the neuroproteins of the Chamorro people [3,4,9-11]. BMAA has been shown to be widespread in a diverse array in addition to the Pacific islands [8,12,13]. This neurotoxic amino acid is produced by cyanobacteria and has been found in marine areas with cyanobacterial blooms in different continents including Europe and North America and is considered to bioaccumulate from invertebrates to animals of higher trophic levels, ending in human consumption [14-16]. In agreement with that, the brains of ALS patients may contain a significant amount of BMAA, strengthening the link for this toxin acting as an environmental trigger together with genetic factors for developing ALS [4]. However, in Europe and North America, the dietary source, if any, remains in dispute but Field et al. recently reported significant concentrations of BMAA, in crabs similar to those consumed by individuals in a putative ALS cluster in Chesapeake Bay, USA [17]. Such ALS clusters are useful tools for studying potential environmental exposures and the Guam cluster is probably the most investigated one in the world. The Hérault district, in southern France, has a large marine coast, with known cyanobacterial blooms in different lagoons. We thus searched for ALS clusters in the whole district area. 

## Materials and Methods

### Ethics

The present study was submitted to the ethics committee “CPP sud-méditerranée IV”. In his written report (Q-2013-09-05), the ethics committee issued a formal written waiver for the need of ethics approval and stated that the procedure did not require patients’ information or consent. 

### ALS patients

Incident ALS cases diagnosed at the ALS center of Montpellier (the capital of Hérault district) between 1994 and 2009, were selected from our database if they had a diagnosis of definite or probable ALS, following international ALS criteria [18] and lived in the district. In the ALS center, according to national recommendations, patients were examined quarterly until death, allowing diagnosis to be ascertained with the highest degree of probability. No post mortem tissues were collected from these patients.

### Cluster analysis

ALS clusters were searched for in the Hérault district that represented 1,044,558 inhabitants in 2010, and between 1994 and 2009, corresponded to an accumulated population of 15,061,970 person-years. A cluster is defined as an abnormal grouping, in time or space, either of illnesses, symptoms or health-related events in a defined population [19]. The standardized incidence ratio (SIR), the only indicator for measuring incidence, is based on the comparison between the total number of observed cases and the number of expected cases in the population area The number of expected cases corresponded to the following formula: incidence in the whole district for a given period of time x the population in the area of interest/100 000. The population of a given area was obtained using public data (INSEE) for years 1990, 1999 and 2009, periods of official census. For each year, a linear interpolation allowed to estimate the population for every town in the district. For each analysis, a spatial unit was considered if its SIR was significantly higher than its reference population (SIR with a 95% CI >1). For the analysis of the cluster, we adopted the Kulldorf’s statistic [20] allowing spatio-temporal aggregations to be identified, subsequently validated using a Monte Carlo test. Then a Stone's test was performed to analyze the changes of relative risks around our putative source, Cluster 1 [21]. The spatial analysis protocol can be used with Geographic System Information (GIS), vectorial or raster software. For the referential statistics of Kulldorf, we used the software Satscan (www.satscan.org). For the Stone test, we used the softwares R (www.r-project.org/) and Qgis with the plus-in Sextante (www.qgis.org/).

### Study site and sampling of water and bivalves

The Mediterranean Thau lagoon is a bivalve farming area that accounts for about 10% of French oyster *Crassostrea gigas* production. Thau lagoon also traditionally supports a high biomass of cultivated mussels *Mytilus galloprovincialis* [22]. 

Thau lagoon is 19 km long, 4.5 km wide and 5 m deep on average. Shellfish are cultured in 3 areas of the lagoon, which together cover about 20% of the total surface. The lagoon is almost closed, with only narrow connections to the Mediterranean Sea through a channel and other small connections that are negligible in terms of water exchange. The catchment area of the lagoon is about 300 km^2^, including land used for agriculture, industrial activities and urban development.

Water samples were collected both inside and outside the bivalve farming area, 26 times between 2 March 2009 and 15 February 2010 [23]. About 5 L of water was collected and pre-filtered through a 200 μm screen to remove any large zooplankton or algal debris. 

Market-size oysters held in the Thau lagoon were collected on 2009/04/27, 2009/06/22, 2009/07/06, 2009/07/27 and 2009/08/24 (see Pernet et al., 2012 for further details about animal maintenance). Mussels were collected by the Ifremer RNO network (Réseau National d’Observation de la qualité du milieu marin) from 1995 through 2008. As the Thau lagoon is a public area, no specific permissions were required for the collections. Moreover, Ifremer, responsible for the collections in the present study, is a public institute with the missions to conduct and promote fundamental and applied researches on marine areas. Mussels and oysters are not endangered or protected species.

### Picophytoplankton flow cytometric determination

Duplicate 1 -ml water samples were preserved in 2 -ml cryovials with 2% (w/v) formaldehyde, after which they were quickly frozen and stored in liquid nitrogen. Picophytoplankton abundances were evaluated using a FACS Calibur flow cytometer (Beckton-Dickinson) fitted with a 488nm laser. Picoeukaryotes and phycocyanin-rich picocyanobacteria were distinguished on the basis of light diffraction (Forward Scatter, FS, related to cell size) and red fluorescence emissions (Chl*a* wave-length > 650 nm). Populations of phycoerythrin-rich picocyanobacteria were identified by their orange fluorescence emissions (phycoerythrin, 542-585 nm). Data were logged using CellQuest software and analyzed with Attractors software (Beckton-Dickinson, Inc., USA). 

### Biochemistry

BMAA was synthesized by Dr. Peter Nunn and compared to an authenticated standard (Sigma B-107 St. Louis, MO). L-2,4-diaminobutryic acid dihydrochloride (DAB-32830), acetonitrile (LC-MS Chromasolv, 34967) was purchased from Sigma-Aldrich (St. Louis, MO). Formic acid was purchased from Thermo Scientific (28905). Water (W-6-4 Optima LC/MS) was purchased from Fisher Scientific. Negative controls included *Lycopersicum esculentum, Saccharomyces cerevisiae* and derivatized blanks. 

Each sample was hydrolyzed in 6.0 M HCl and heated for 16 hrs at 110°C. Samples were filtered through a 0.22 μm centrifugal filter device (Millipore UltrafreeMC, 14,000 x g for 3 min, Labnet-Specrafuge 16M) and dried to complete dryness using a Thermo-Savant SC250DDA Speed Vac Plus (Waltham, MA). Samples were resuspended in 20mM HCl and appropriately diluted for a balanced reaction and derivatized with 6-aminoquinolyl-N-hydroxysuccinimidyl carbamate (AQC Waters AccQTag reagent, PN WAT052880). Samples were injected into a triple quadrupole, - MS/MS instrument (Thermo Scientific Finnigan TSQ Quantum UltraAM, San Jose, CA) after separation with an Ultra High Pressure Liquid Chromatography (Waters Acquity-UHPLC) system with a Binary Solvent Manager, Sample Manager and a Waters AccQTag Ultra column (part# 186003837, 2.1x100 mm) at 55°C.

Separation was achieved using gradient elution at 0.65 ml/min in aqueous 0.1% (v/v) formic acid (Eluent A) and 0.1% (v/v) formic acid in acetonitrile (Eluent B): 0.0 min= 99.1% A; 0.5 min= 99.1% A curve 6; 2 min= 95% A curve 6; 3 min= 95% A curve 6; 5.5 min= 90% A curve 8; 6 min= 15% A curve 6; 6.5 min =15% A curve 6; 6.6 min=99.1% A curve 6; 8 min =99.1% A curve 6. Nitrogen gas was supplied to the heated electrospray ionization (H-ESI) probe with a nebulizing pressure of 40 psi and a vaporizer temperature of 400°C. The mass spectrometer was operated under the following conditions: the capillary temperature was set at 270°C, capillary offset of 35, tube lens offset of 110, auxiliary gas pressure of 35, spray voltage 3500, source collision energy of 0, and multiplier voltage of -1654. A divert valve was used to deflect flow during the beginning and end of the gradient. The second quadrupole was pressurized to 1.0 mTorr with 100% argon. Product-ion analysis of BMAA used 459 *m/z* as the precursor ion for collision-induced dissociation (CID) and thereby all other ions were excluded in the first quadrupole. Further two-step mass filtering was performed during selective reaction monitoring (SRM) of BMAA after CID in the second quadrupole, monitoring the following transitions: *m/z* 459 to 119, CE 21 eV; 459 to 188 CE 38 eV; 459 to 214 CE 35 eV 459 to 258 CE 21 eV; 459 to 289 CE 17 eV; 459 to 171 CE 38 eV (24). The resultant product ions were detected, after passing the third quadrupole and their relative abundances were quantified. 

Detection limits and limits of quantification of BMAA and DAB, a neurotoxic isomer of BMAA, were determined experimentally by injecting dilution series of authenticated stock solutions at 4 concentrations (0.015, 0.15, 1.5, 10.5 μg/L). The intra-day and inter-day precisions were evaluated according to the percent relative standard deviation (RSD) for peak area, retention time and SRM transitions ratio. HPLC, UPLC-UV, and UPLC-MS data using AQC derivatization and following Banack et al. [24] were used to confirm results. Samples were run blinded to the instrument operator as well as the lab staff.

## Results

### Cluster analysis

A total of 381 patients fulfilled diagnostic criteria of probable or definite ALS in the study period (between January 1994 and December 2009).The incidence rate of ALS in the Hérault district was thus 2.53 per 100,000 inhabitants per year of follow up.

**Figure 1:**
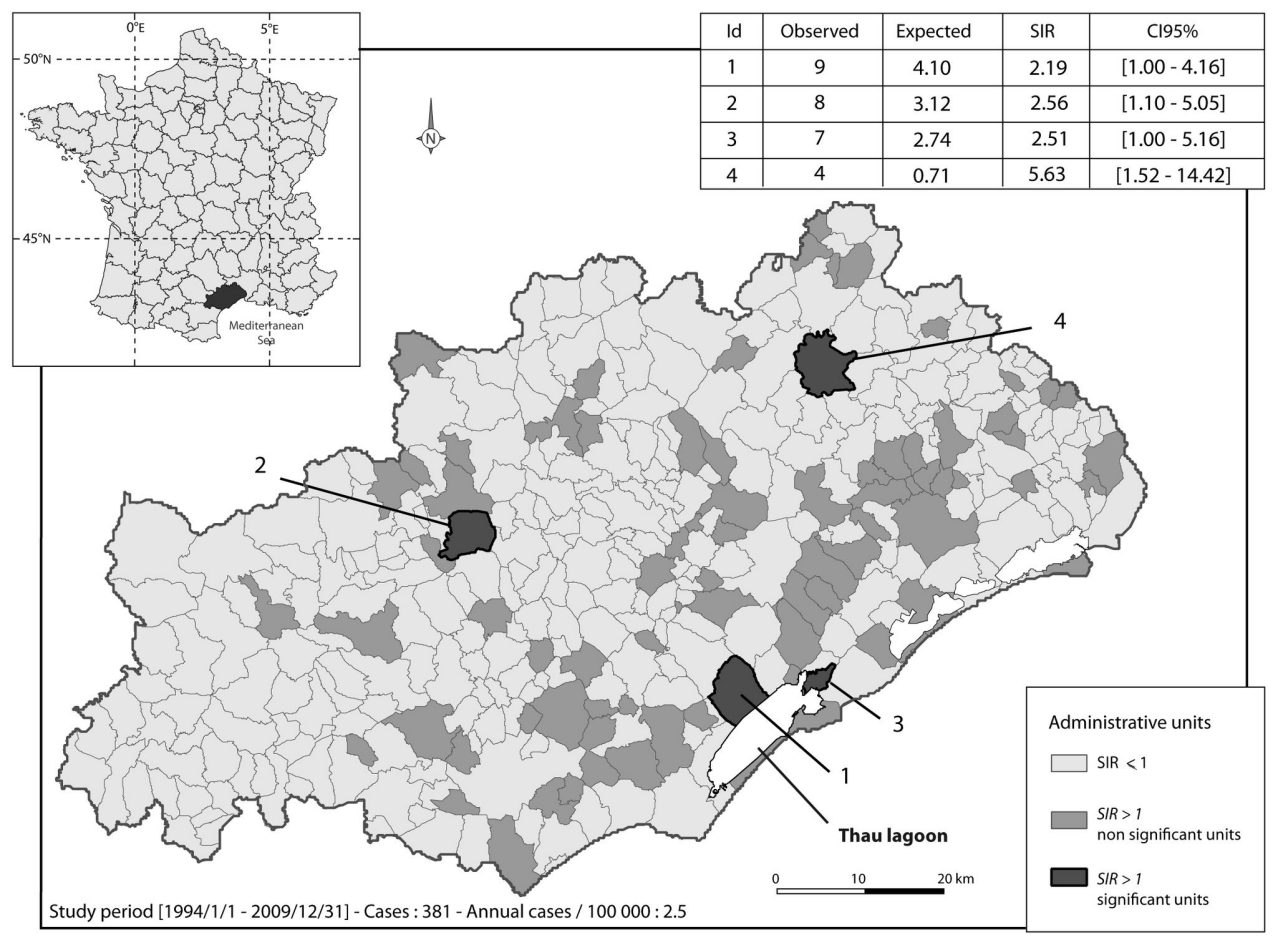
Four spatial units with a significant SIR. The Hérault district is a coastal area in the very southern France, surrounding the mediterranean sea. The four significant spatial units are featured in dark grey. Two spatial units, cluster 1 and 2, surround the Thau lagoon.

**Figure 2:**
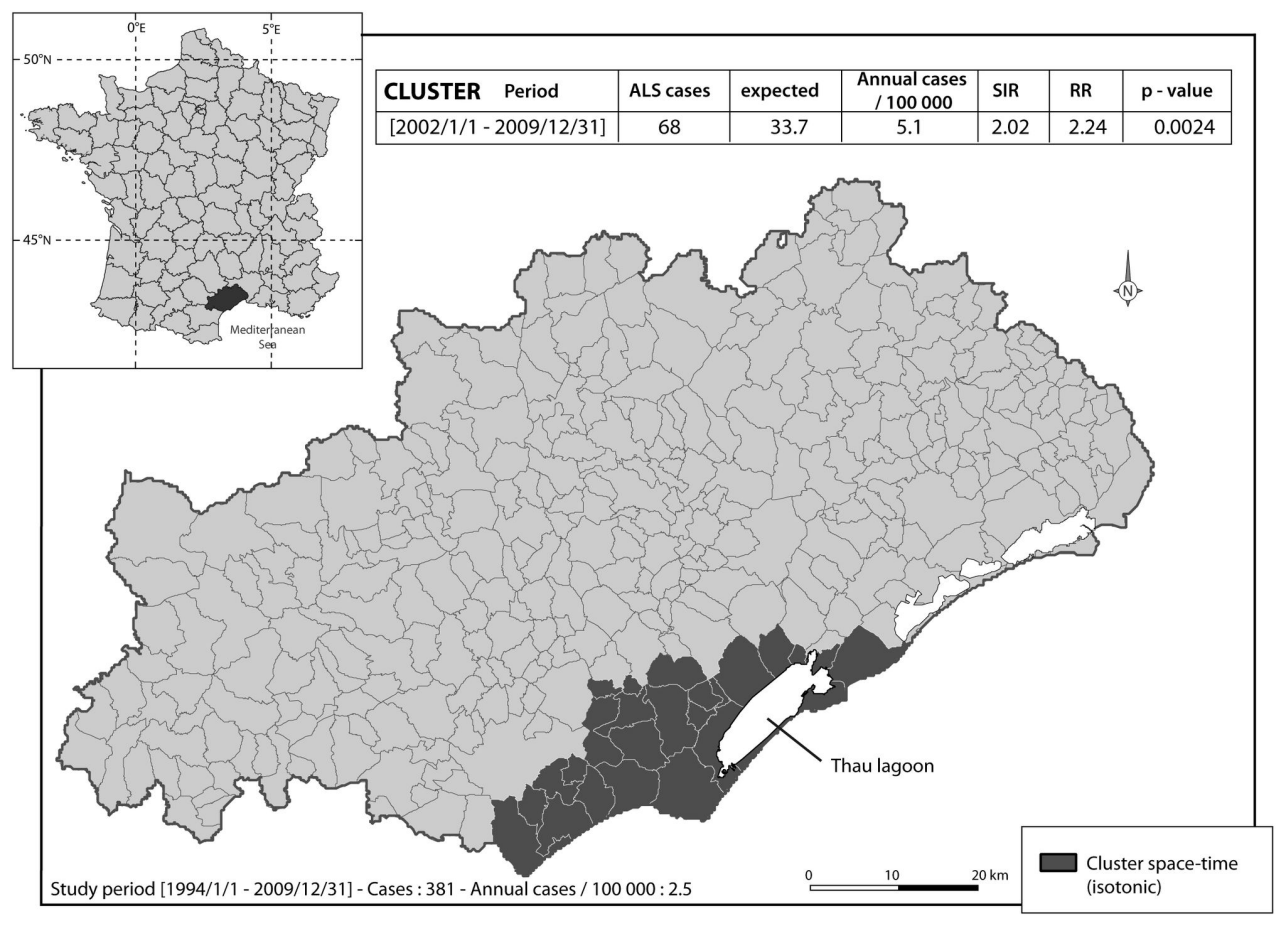
Spatio-temporal ALS cluster in the Hérault district. This cluster (dark grey area) is composed of 26 townships and has been identified for the period between January 1992 and December 2009, with 68 ALS cases for 33.7 expected (SIR = 2.02, RR = 2.24, p = 0.0024).

**Figure 3:**
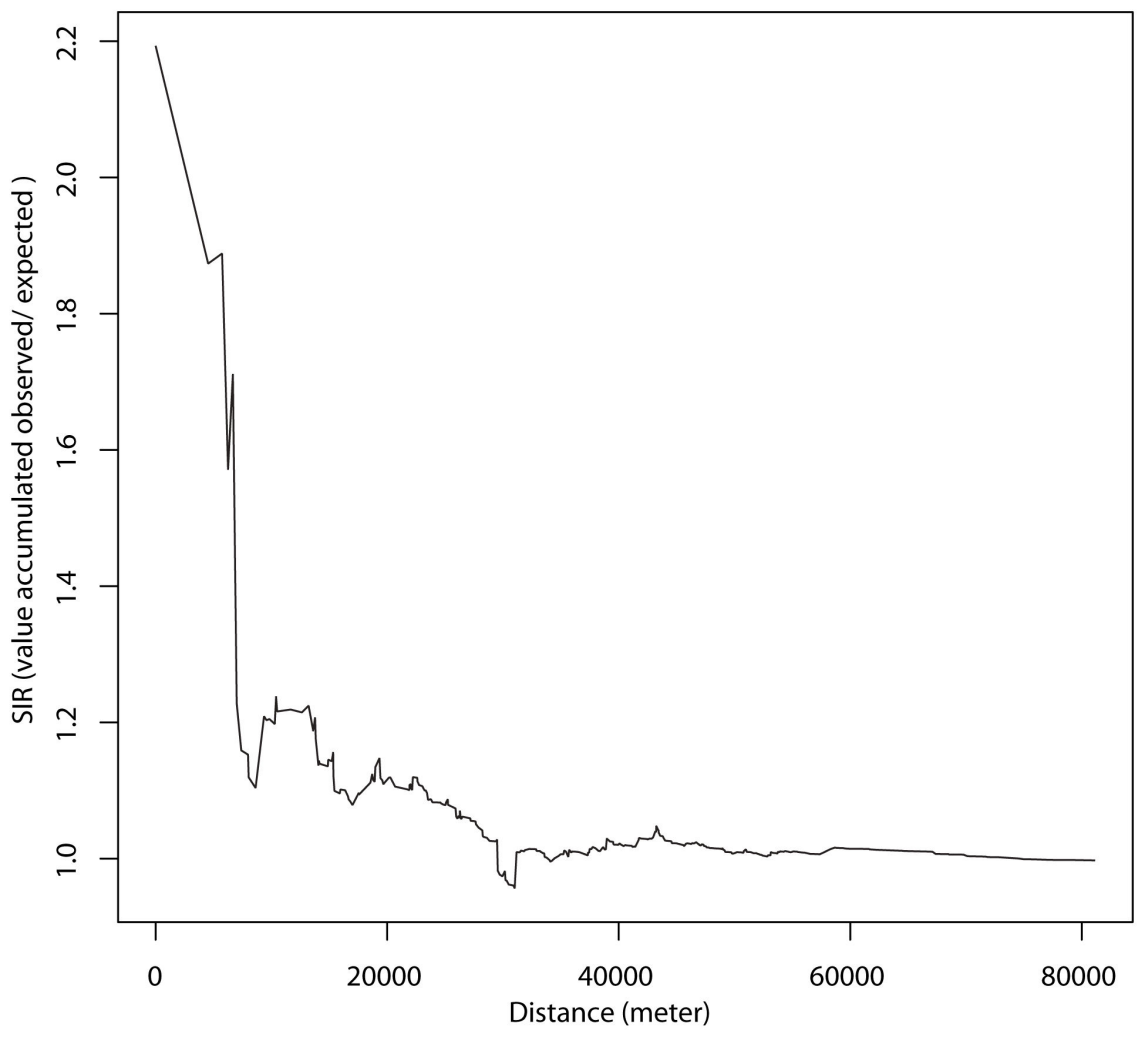
SIR evolution in the Hérault district according to the distance from cluster 1. Maximum SIR is noted in cluster 1 and decreases rapidly when increasing the distance from this epicentre. Significance is p = 0.025, Stone's test for raised incidence around locations. Type of boots: parametric. Model used when sampling: Poisson. Number of simulations: 999. Statistic: 2.19325.

In a first step, cluster analysis identified four spatial units with a significant SIR (Figure 1). Two of them corresponded to towns surrounding the Thau lagoon. The first one, cluster 1, had a SIR of 2.18 [1.00-4.16] with 9 cases observed as compared to the 4.1 cases expected. Cluster 2 had a SIR of 2.51 [1.00-5.16] with 7 cases observed compared to the 2.74 expected. The last two clusters, non-coastal ones, namely cluster 3 and 4, had a SIR of 2.56 [1.10 - 5.05] and 5.63 [1.52-14.42], respectively. In a second step ALS cluster analysis searched for geographical aggregations that significantly comprised a large number of cases. In a logic of aggregation of aforementioned elementary spatial units, we identified one spatio-temporal ALS cluster in the Hérault district area. It was composed of twenty-six townships close to the coast, almost all edging the Thau Lagoon (Figure 2). This aggregation has been detected for the period between 2002/1/1 and 2009/31/1, corresponding to 1,247,704 persons exposed during this period. With 68 cases observed compared to the 33.7 expected, the incidence rates in this area and for this period were 5.1 per 100,000 inhabitants per year of follow up, presenting a SIR of 2.02 and a RR = 2.24 (p= 0.0024). The Stone test was positive, showing that the closer to cluster 1 the higher the significance (p = 0.025, Figure 3). As it has already been described that BMAA may be present in marine areas, we aimed to determine whether the littoral location of cluster 1 could account for the existence of the focus. The cluster analysis was then performed according to the different morphologic wholes in the district and it was negative. It was the same when taking into account the geographical characteristics (district of regional development, enhancement of soils etc.) of the whole district. 

A clustering of disease may be due to chance, genetics or environmental conditions, or a combination of both genetics and environment. The strength of the previous statistical approach led us to consider the two latter aspects, even if a chance association is difficult to definitively rule out. The genealogy of all the ALS cases in cluster 1 was done and no case of familial ALS could be found. Moreover, we searched for common ancestors on three generations between the patients in this area. Once again, no apparent common ancestry could be found. Because of the lack of familial relationships between ALS patients, a genetic analysis to search for the known mutations of familial ALS in the population of cluster 1 was not performed.

In the Thau lagoon area, food intake as well as the economic structure in focused on shellfish culture, mainly oysters and mussels. We thus analyzed the marine biological environment by serial collections of water and shellfish, and tested it for the presence of BMAA.

### Picophytoplankton composition and abundance

**Figure 4:**
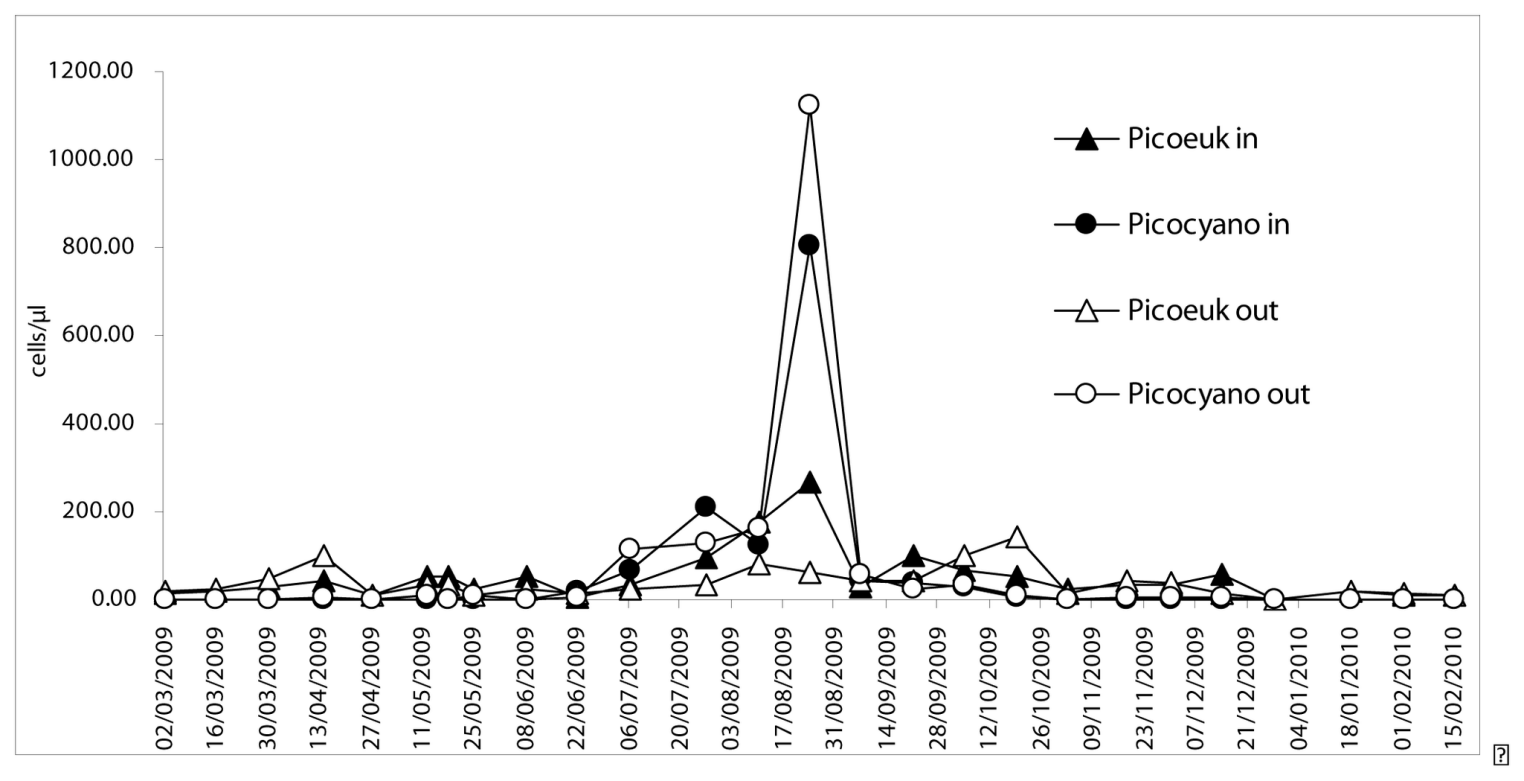
Seasonal variations of the picophytoplankton community in Thau lagoon from March 2009 until February 2010. Circles: picocyanobacteria; triangle: picoeukaryotes; black: inside the farming zone, open: outside the farming zone.

Flow cytometry analysis showed that picoeukaryotes and phycoerythrin-rich (PE-rich) picocyanobacteria were the two components of the picophytoplankton community (Figure 4). Picoeukaryotes were present all year long while PE-rich cyanobacteria were detected from June to October 2009. The highest abundances of the picocyanobacteria, which is potentially the main source of BMAA within this phytoplankton community, were recorded in July and August (respectively 2.10^8^ cells/l and 8.10^8^ cells/l, Figure 4). For both picoeucaryotes and PE-rich cyanobacteria, the abundances were identical within and outside the farming zone.

### Biochemistry

Method validation for BMAA determination, indicated that concentrations of both BMAA and DAB were linear across the range examined (BMAA f(x) = 31887463155463x +1912, R_2_=0.99; DAB f(x) = 104331661365930x – 205199, R_2_= 1) and the results were repeatable. Mean intra-day peak area for the *m/z* 459 to *m/z* 171 transition of 1.5 μg/L concentration of BMAA was 3018539, 6.3% RSD. Mean peak area for the *m/z* 459 to *m/z* 171 transition of 1.5 μg/L concentration of DAB was 2026304, 6.4% RSD. BMAA ratios of the *m/z* 289 and *m/z* 119 relative to *m/z* 171 were 29.9% and 16.7% respectively. DAB ratios of the *m/z* 289 and *m/z* 119 relative to *m/z* 171 were 5.9% and 5.0% respectively. BMAA and DAB were baseline resolved (retention time: 4.98 min L-BMAA, 5.13 min DAB; resolution 3.3 min). The limit of detection (LOD) was 0.0065 and 0.0013 picomoles per injection for L-BMAA and DAB respectively. Limit of quantification (LOQ) was 0.013 picomoles per injection for both L-BMAA and DAB. 

All shellfish were collected from a costal lagoon known to have annual cyanobacterial blooms, and all shellfish samples were blinded prior to analysis. Both BMAA and its neurotoxic isomer DAB were detected in the shellfish samples examined (Table 1 and 2). Overall, mussels collected between 1995 and 2008 showed higher BMAA concentrations than oysters collected during the spring and the summer 2009 (mussels mean 3.3 ± 1.4 μg/g; oysters mean 1.3 ± 0.19 μg/g) but the DAB concentrations did not differ between the two shellfish types (mussels mean 1.1 ± 0.35 μg/g; oysters mean 1.3 ± 0.19 μg/g). 

**Table 1:** BMAA and DAB concentrations in mussels.

Date	BMAA (µg/g)	DAB (µg/g)
22/AUG/1995	6.0	1.3
28/AUG/2000	4.5	1.0
12/AUG/2002	2.9	1.0
24/NOV/2003	1.8	0.7
29/NOV/2004	2.5	0.9
4/DEC/2007	2.5	1.2
12/NOV/2008	3.1	1.8

**Table 2:** BMAA and DAB concentrations in oysters.

Date	BMAA (µg/g)	*SD*	DAB (µg/g)	*SD*
2009/APR/27	0.6	0.07	0.9	0.41
2009/JUN/22	1.6	0.14	1.1	0.21
2009/JUL/06	1.6	0.82	1.8	0.60
2009/JUL/27	1.5	0.49	1.6.	0.65
2009/AUG/24	1.4	0.39	1.4	0.64

n = 3 for each row date ; SD: standard deviation

Concentrations of BMAA in mussels and oysters were the highest during the summer, which coincide with blooms of picoplankton. For instance, in mussels, BMAA concentrations were 4.45 ± 1.55 µg/g during summer and 2.48 ± 0.53 µg/g in autumn and winter, and in oysters concentrations were 1.53 ± 0.10 µg/g in summer vs. 0.60 µg/g in April. Such seasonal variation was not observed for the concentrations of the BMAA isomer DAB in either mussels or oysters. 

## Discussion

We identified a significant and large ALS cluster in the Hérault district in Southern France, surrounding the Thau lagoon area, the main shellfish production area of the French Mediterranean coast in which human populations are major consumers, all year long, of local seafood (molluscs and crustaceans). BMAA was identified both in mussels and oysters held in this lagoon and the toxin concentrations (measured blind) were the highest in oysters during the summer when cyanobacteria blooms, considered as the source of BMAA, largely occur. In the Thau lagoon, blooms of picocyanobacteria have become more prominent during the last twenty years, likely reflecting a gradual decrease in nutrient inputs from the watershed and changes in hydro-climatic conditions [25]. These authors showed that most of these picocyanobacteria were of the genus *Synechococcus*, as identified by electron microscopy. Interestingly, BMAA had previously been detected in a strain belonging to this genus [26]. Although *Synechococcus* cells are too small (ca. 0.52 µm^3^) to be directly filtered by oysters or mussels, heterotrophic nanoflagellates and ciliates, which are the major picoplankton grazers consumed by bivalves could act as a carrier of BMAA-rich picocyanobacteria [27-30]. Our study is the first to analyse BMAA in mussel specimens collected almost 20 years ago. These analyses suggest that detection of significant amounts of BMAA in bivalves held in the Thau lagoon is not a novel phenomenon, as marine picocyanobacteria blooms are commom.

Although several confounding factors prevent us from direct comparison of BMAA concentrations between mussels and oysters collected in this study, it is noteworthy that the highest BMAA concentrations were observed in mussels. These differences are in good agreement with interspecific differences in physiological rates. Indeed, the filtration activity of bivalves, which is measured by the rate of clearance, i.g. the volume of water cleared of particles per unit of time per dry weight of bivalve, is generally higher in mussels than in oysters [31-33]. 

While human populations in the ALS cluster are major consumers, all year long, of local seafood, this may not be the only possible way of contamination. It has been proposed that aerosolization of cyanobacteria or BMAA could be a candidate mode of intoxication for the Gulf war veterans [8]. In the marine area of Thau lagoon marine winds frequently blow strongly towards the coast and may expose people to the risk of aerosolization. However, it seems difficult to suggest that this mode of contamination, alone, could be the explanation of the cluster, for at least two reasons. First, throughout the year, cyanobacteria concentrations (<5000 cell/ml) seem too low to have a direct impact via aerosolization, even if cyanobacteria abundances are much more important during summer. Secondly, in the case of aerosolization, no particular biomagnification will occur so that the quantities of cyanobacteria or BMAA needed would probably be too important to be reached by this way alone.

Cluster 1 was the only significant ALS cluster arising from our study. The incidence of ALS in this cluster was more than twice the estimated incidence of ALS in France and in the Hérault district. For this cluster study, we did not perform a capture-recapture analysis to improve the recruitment of ALS cases. We cannot thus exclude, however, that, using a more exhaustive estimation of the number of ALS cases, other clusters could be identified. In the search for potential causes to the cluster, a systematic enquiry about familial antecedents of ALS and a study of genealogy did not identify familial ALS cases nor a common ancestry between the patients. Our focus on shellfish was due to the well-known existence of shellfish farms close to cluster 1 as well as the significantly high quantities of shellfish the local population eats every day and all year long. On the other hand, the use of a questionnaire would have helped obtaining well documented habits, recreational activities or diets. Such a questionnaire, not available at the time of patients’ follow up, is now being used for the prospective approach of this cluster area. As already described for ALS clusters in Limousin, we nevertheless searched for other exposures in the ALS cluster area and uncovered the existence of several companies treating or producing wood products [34]. Such industrial activities may be responsible for phosphorus effluents in the water as well as increased human exposure to solvents from glues, known to contain formaldehyde, a solvent already implicated in increased ALS risk [35]. However, the study of the geographical distribution of these companies showed that they were spread all along the area but were not particularly concentrated in cluster 1 area (data not shown). Another pathway to consider is the existence in the area of intensive agriculture, an activity known to use pesticides. One cannot exclude a conjugated action of such molecules with BMAA on the neurodegenerative process [36].

## Conclusion

In Europe and USA, the cyanotoxin BMAA has been found into marine areas and is suspected to be magnified through the food chain potentially resulting in human consumption. We described here a dietary exposure to the cyanotoxin BMAA in the ALS cluster of Hérault, in southern France. While it is not possible to demonstrate a direct link between the presence of BMAA in shellfish and the higher incidence of ALS in our cluster, we consider that this work adds further data regarding public health concerns about the potential role of the dietary exposure to the cyanotoxin BMAA in sporadic ALS.
